# Feasibility & Preliminary Efficacy of Structured Programming and a Parent Intervention to Mitigate Accelerated Summer BMI Gain: A pilot study

**DOI:** 10.21203/rs.3.rs-1466063/v1

**Published:** 2022-03-29

**Authors:** R Glenn Weaver, Bridget Armstrong, Elizabeth Adams, Michael Beets, James White, Kate Flory, Dawn Wilson, Alex Mclain, Brianna Tennie

**Affiliations:** University of South Carolina; University of South Carolina Arnold School of Public Health; University of South Carolina Arnold School of Public Health; University of South Carolina Arnold School of Public Health; University of South Carolina Arnold School of Public Health; University of South Carolina Department of Psychology; University of South Carolina Department of Psychology; University of South Carolina Arnold School of Public Health; University of South Carolina Arnold School of Public Health

**Keywords:** Children, overweight, obesity, intervention

## Abstract

**Background:**

This study assessed initial feasibility and preliminary efficacy of providing children a free summer day camp and a parent intervention to improve self-regulation and mitigate accelerated summer BMI gain.

**Methods:**

This pilot 2×2 factorial randomized control trial used a mixed methods design to evaluate providing children a free summer day camp (SCV), a parent intervention (PI), and the combination of these two strategies (SCV + PI) to mitigate accelerated summer body mass index (BMI) gain. Feasibility (i.e., recruitment capability, retention, compliance, treatment fidelity, acceptability) was examined using means, standard deviations, and percentages for relevant variables. Changes in BMI were estimated using intent-to-treat and post-hoc dose response analyses via multilevel mixed effects regressions.

**Results:**

A total of 89 families participated, with 24 participants randomized to the PI group, 21 randomized to the SCV group, 23 randomized to the SCV + PI group, and 21 randomized to the control. Parents and children found the summer program acceptable but attendance at the summer program and engagement in the PI were low due to COVID-19 and lack of transportation. Intent-to-treat analyses showed no statistically significant difference between groups in summer BMI gain. Post-hoc dose response analyses showed that for each day (0 to 29) of summer programming children attended they gained − 0.009 (95CI= −0.018, −0.001) less in BMI z-score.

**Conclusions:**

Engagement in both the SCV and PI was not ideal and was likely due to COVID-19 and lack of transportation. Providing children with structured summer programming to mitigate accelerated summer BMI gain may be an effective strategy. Thus, a larger trial may be warranted, but more work is needed to ensure children attend the programming.

Trial registration: The trial reported herein was prospectively registered at clinicaltrials.gov. Trial #:NCT04608188.

## Background

For all children, summer represents a “window of vulnerability” in which body mass index (BMI) gain occurs at an accelerated rate compared to the school year (1, 2). Moreover, excessive BMI gain during summer is more pronounced in children from low-income(3) and traditionally minoritized households (4). The structured days hypothesis posits that accelerated summer BMI gain may occur because children engage in higher levels of obesogenic behaviors (e.g., watching screens, eating junk foods) during summer, when they are exposed to days that are less structured (5). Further, parents may relax rules and routines that provide structure in the home (e.g., set bedtimes and mealtimes) during the summer.

Preliminary research on children’s obesogenic behaviors over summer suggests that sedentary behaviors increase and moderate-to-vigorous physical activity (MVPA) decreases while sleep shifts later and becomes more variable (6–9). A recent natural experiment provides evidence this may be due to the removal of the school day during summer (10). The recent Novel SARS-CoV-2 (COVID-19) pandemic-related school closures provide further compelling evidence of the protective effect of the in-person school day for children’s obesogenic behaviors. A large body of evidence suggests that the closure of schools had a negative impact on children’s diet (11, 12), sleep (13, 14), physical activity (14, 15), and sedentary behaviors (11, 14), and that this corresponded to accelerated BMI gain in children around the world (16–18).

An under-researched, but potentially important factor in this work, is the role of self-regulation. Routines embedded within structured days may promote children’s ability to self-regulate, the capacity to monitor and control one’s thoughts and emotions to meet the demands of a situation (19, 20). Self-regulation may be a key mechanism for maintaining a healthy weight as poor self-regulation in early childhood is linked to overweight and obesity later in life (21, 22). Studies also show that parents are key influences on children’s obesogenic behaviors (23). Rules and routines instituted at home can also lead to relatively more structured days which may increase a child’s ability to self-regulate. Thus, it may be crucial to target parents in interventions aiming to improve children’s self-regulation and mitigate accelerated summer BMI gain.

Summer day camps (e.g., 7AM-5PM, 8–10wks) are a setting that can provide children a structured, healthy environment, during the summer. For instance, a growing number of summer camps participate in the United States Department of Agriculture Summer Food Service Program, which sets nutritional guidelines related to food quantity and quality (24). Attendance at camps can help regulate sleep schedules because of camp start times (e.g., 7–9am), and children attending summer day camps accumulate between 60 and 90 minutes of moderate-to-vigorous physical activity each day (25, 26). However, most children from low-income and minoritized households have limited access to structured environments during the summer because typical summer camp costs $288 per week to attend (27). This cost is prohibitive for many low-income families, with only 20% of children attending summer camps coming from these families (27). Providing access to existing community-operated camps during the summer has the potential to lead to marked improvements in the obesogenic behaviors and weight of children from low-income households over the summer.

The purpose of this pilot study was therefore, to test the initial feasibility and preliminary efficacy of providing children from low-income communities with vouchers to attend a summer day camp, a parent intervention targeting goal setting and behavioral self-monitoring, and the combination of these two strategies for the purpose of improving self-regulation and mitigating accelerated summer BMI gain.

## Methods

### Study Design and Setting

This pilot 2×2 factorial RCT was prospectively registered in clinicaltrials.gov #NCT04608188. The study was conducted in one primary school in a southeastern state of the U.S. during the summer between the 2020–21 and 2021–22 school years. The participating school was selected for three reasons. First, the school served the target population of elementary children, and was sufficiently large to meet the study’s recruitment goals (i.e., 535 students). Second, students served were predominantly from minoritized families that were low-income (81% minority, 90% of families in poverty). Finally, the school did not previously offer a summer day camp on campus. This ensured that the likelihood of children in the control condition attending a summer day camp was low.

### Sample Size Considerations

The power analysis, performed using G*Power (v.3.1.7), determined that the study was sufficiently powered to detect a Cohens d effect size of 0.20, assuming a sample size of 80 participants, power of 0.80, correlation between measures of 0.80, and two-sided alpha of 0.05. This was deemed sufficient based on past large-scale longitudinal studies of school year vs. summer weight gain (1, 28), and studies that have provided a structured program to children during the summer (29–31). Further, a sample of 80 participants was chosen so as to be sufficiently large to evaluate metrics of feasibility.

### Participant Recruitment

**Figure 1:**
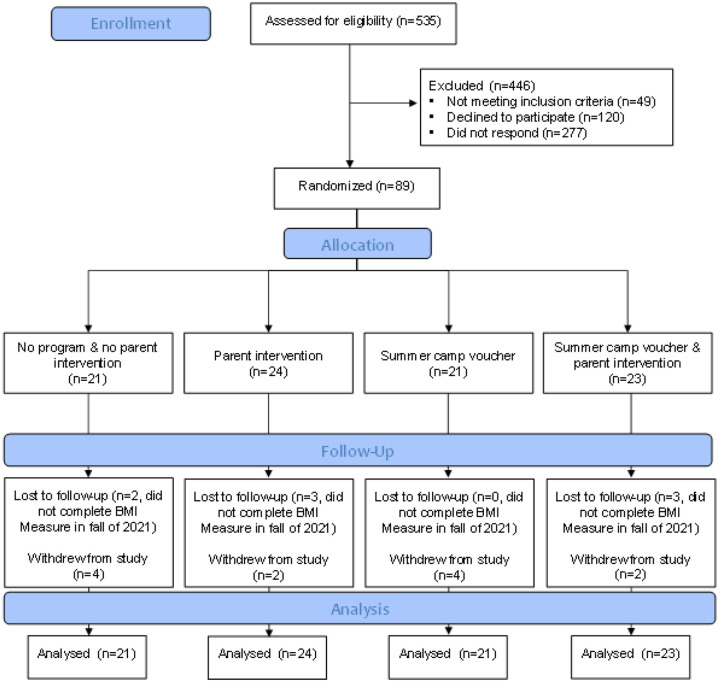
Consort Flow Diagram of Participants Through the Study

Prior to enrollment of the first participant, the study protocols were approved by the first author’s institutional review board. Inclusion criteria were that children were in the K-4th grade at the participating school. Exclusion criteria for participation was a physical disability that limited physical activity (e.g., wheelchair use, visual impairment), and/or plans to enroll in a summer camp program during the study summer. See Figure 1 for the Consort flow diagram of participants. Eligible children were recruited to participate in the study in the spring of 2021 via informational fliers and consent forms sent home from school. Signed consent forms were returned to the school and collected by trained research assistants. Verbal assent was obtained from children prior to each measurement occasion.

### Participant Allocation

Participants in the study were randomly allocated to one of four conditions: 1) control, 2) receive a summer day camp voucher (SCV), 3) receive a parent intervention (PI) targeting goal setting and behavioral self-monitoring, or 4) receive a summer day camp voucher and a parent intervention (SCV+PI). Because some families had siblings participating in the study, randomization was completed at the family level leading to slightly different numbers of participants in each group. The allocation was conducted by a statistician independent of the study team. Randomization was completed following baseline data collection using the runiform command in Stata (v16.1, College Station, TX). Blinding of participants and study staff to participant condition was not possible in this trial.

### Intervention

#### Summer day camp voucher.

The voucher covered enrollment fees associated with accessing a summer day camp operated at the participating school by the local Boys & Girls Club Program and transportation via bus to and from the camp. The summer day camp was not singularly focused, such as sport camps or academic-only camps. Rather, the camp provided indoor and outdoor opportunities for children to be physically active each day, provided enrichment and academic programming, as well as provided breakfast, lunch, and snacks. Importantly, the camp was enrolled in the United States Department of Agriculture Summer Food Service Program. Thus, all meals adhered to the Summer Food Service Program nutrition guidelines. Complete details of the Summer Food Service Program meal patterns can be found here: https://www.fns.usda.gov/sfsp/meal-patterns. The camp opened at 7:30am and closed at 5:30pm and was designed to operate daily (Mon-Fri) for 8 weeks during the summer. Physical activity opportunities were scheduled for 3 to 4 hours each day, with the remaining 4 to 5 hours dedicated to enrichment/academics or meals/snacks. The camp operated according to Boys & Girls Club routine practice, with no outside assistance from the investigative team.

#### Parent intervention.

The PI was founded on goal setting (32, 33) and behavioral monitoring of their child’s sleep and physical activity (34), which are key components that underly effective behavioral interventions (35). In May, children received a Fitbit to wear throughout the summer. After one week of observational wear each child’s physical activity and sleep data were downloaded via Fitabase. Children’s parents were then contacted to set up a 20-minute goal setting phone call with a member of the research team. On this call, parents were provided with their child’s physical activity and sleep data for the week prior and the data was situated within the physical activity (MVPA—>60 minutes/day)(36) and sleep guidelines (9–12 hours/night)(37) for children. On this phone call a trained interventionist worked with parents to set goals surrounding the child’s physical activity and sleep using a standardized goal setting worksheet. Following the initial goal setting phone call children’s physical activity and sleep data continued to be downloaded via Fitabase each week. From this data the number of days that the child met physical activity and sleep guidelines were distilled and tailored messages were texted to parents once per week which provided feedback on the number of days that their child met physical activity and sleep guidelines and how this complied with their goals. For those meeting their goals an encouraging message was sent (e.g., Awesome week for (child name)! Meeting the National Sleep Foundation recommendation of 9–11 hours every night helps (child name) to stay focused & work hard.). For those not meeting their goals a brief message with strategies to meet behavioral guidelines was sent (e.g., Children, like (child name) need 9–11 hours of sleep every night. Try setting an early enough bedtime so they can wake up energized everyday!).

### Feasibility Outcomes

The feasibility outcomes and their definitions can be found in Table 1. These outcomes and their definitions were identified and adapted from literature on pilot studies from the National Institutes of Health (38), implementation frameworks (39, 40), and other literature on implementation research and pilot studies (41, 42). Implementation outcomes included recruitment capability retention, compliance, treatment fidelity, and acceptability.

### Efficacy Outcomes

#### Body Mass Index.

Using a portable stadiometer (Model S100, Ayrton Corp., Prior Lake, Minn.) and digital scale (Healthometer model 500KL, Health o meter, McCook, Ill.), children’s heights (nearest 0.1 cm) and weights (nearest 0.1 lbs.), without shoes, were collected by trained research assistants in the spring (i.e., May – prior to summer break) and fall (August – following summer break), at their school, during regularly scheduled physical education classes. BMI was calculated (BMI= kg/m^2^) and transformed into age- and sex-specific z-scores (43).

#### Self-regulation.

Self-regulation was captured using three sub-scales on the Teacher Behavior Rating Inventory of Executive Function, Second Edition (44). The inhibit, self-monitor, and emotional control subscales were collected in the spring (i.e., May – prior to summer break) and fall (August – following summer break). The inhibit subscale measures a child’s control impulses and ability to appropriately stop behavior at proper times. The self-monitor subscale assesses a child’s ability to track the effect of their behavior on others. Finally, the emotional control subscale measures a child’s ability to modulate emotional responses appropriately. Teachers completed the survey questions for students that were in their classes during a regularly schedule faculty meeting.

### Deviations Due to the COVID-19 Pandemic

Three deviations in the protocol and study design were necessary due to the COVID-19 pandemic. Reporting of these deviations is guided by the framework for reporting adaptations and modifications to evidence-based interventions (45). The first modification that was made to the study was that free summer programming was made available to children who were struggling academically in the school district, including children in the control and PI only group that would not have had access to free summer programming in past summers. This decision was made by the participating school district in January of 2021 when they received funding via the Coronavirus Aid, Relief, and Economic Security (CARES) Act, to operate expanded programming that was free for children in the district to attend in the summer of 2021. The goal of the programming was to alleviate the learning gaps that grew during the closure of school buildings during COVID-19 pandemic. The second modification that was made was that the summer camp was operated for 6-weeks instead of 8-weeks. This modification was made by the Boys & Girls club program operating the camp due to an abbreviated summer break in the participating school district in the summer of 2021. Typically, summer break is 11 weeks; however, because the school district delayed the start of the 2020–2021 school year the end date of the school year was later and thus the summer was only 8 weeks long. The decision to run an abbreviated camp was made in February of 2021. The third modification that was made was that transportation was not provided to the intervention summer camp. The school district made this decision because they had a shortage of school bus drivers due to the COVID-19 pandemic. This decision was made in May of 2021.

### Analyses

#### Analysis A: Feasibility Evaluation outcomes.

Means, standard deviations, and percentages were computed for all relevant variables for recruitment capability, retention, compliance, and treatment fidelity. Parent, administration, and teacher/staff interviews were uploaded into a single file in QSR NVIVO Version 12 (Sage Publications Software). Two coders coded the data independently using a three-step latent coding technique(46) guided by grounded theory(47) and an immersion crystallization approach (48). Coders first read a single transcript and generated codes by grouping recurring words, phrases, and themes. Coders then met with a third reviewer to review codes, integrate/add codes to a running list of codes generated from each transcript (i.e., coding guide), and to arbitrate any disagreements between coders. Disagreements between coders were resolved via discussion. Finally, coders reread the transcripts to determine if the coding guide had reached saturation (49). This iterative process was repeated until all transcripts were read and a comprehensive coding guide was created. Codes were classified into broad level themes. Themes were developed using inductive analysis. Several steps were taken to ensure the trustworthiness of findings. These include triangulation of the qualitative data, iterative questioning, frequent peer debriefing between coders and a third reviewer, and negative case analysis in the development of themes (50).

#### Analysis B: Efficacy outcomes.

For the efficacy outcomes all inferential analyses were completed in January of 2022 using R version 4.0.3 with α levels set to *P* <0.05 (51). Data were assessed for normality and descriptive statistics of child characteristics and outcome variables were examined at baseline. The analyses were estimated using an intent-to-treat approach (52, 53). Post-hoc dose response analyses were also completed. The decision to complete dose response analyses was made because of the school districts operation of expanded programming that was free for children in the district to attend in the summer of 2021. This led to contamination across groups in summer program attendance. For the intent-to-treat analyses, separate multilevel mixed effects linear regressions, with measures nested within children, were estimated for each outcome (i.e., BMI z-score, inhibit t-score, self-monitor t-score, and emotional control t-score). Models were estimated with dummied group (control, SCV, PI), time (spring prior to summer, fall post-summer), and all group-x-time interactions. For the dose response analyses, models with the same nesting structure and outcomes were estimated with total days attending a summer program (continuous) and total days attending a summer program-x-time interaction included. All analyses included age, sex, and race as covariates in the models. The dose response analyses included group and group-by-time interactions as covariates in the model. Missing data were handled using full information maximum likelihood estimates (54). Finally, for both intent-to-treat and dose response models, sensitivity analyses were estimated with percentage of the median and the 95^th^ percentile of BMI as the outcome. Percentage of the median and the 95^th^ percentile of BMI may be a more appropriate outcome than BMI z-score change for tracking change in age and sex specific BMI over time, especially for those children with extreme BMI z-scores (55–57). These models did not show any differences in magnitude, direction, or statistical significance of effects when compared to the models with BMI z-score as the outcome.

## Results

### Sample Characteristics

The flow of participants through the study is presented in Figure 1. Demographics of participants at baseline are presented in Table 2.

### Feasibility Outcomes

#### Recruitment Capability and Retention.

Recruitment capability and retention outcomes are presented in the Consort flow diagram (Figure 1). In terms of recruitment capability, a total of 535 children were assessed for eligibility with 49 excluded because they were ineligible (not in eligible grade level). Of the 486 eligible 120 (25%) directly declined to participate, 277 (57%) did not return a consent form, and 89 participants (18%) consented. In terms of retention a total of 69 of the 89 participants were retained from baseline to outcome with 15 of 21 (71%) participants retained in the control group, 19 of 24 (78%) retained in the SCV group, 17 of 21 (81%) retained in the PI group, and 18 of 23 (78%) retained in the SCV+PI group.

#### Compliance.

Compliance outcomes are presented in Table 3. A total of 7 of the 21 (33%) children in the SCV group and 11 of the 23 (48%) children in the SCV+PI group attended at least one day of the summer program. Mean attendance for those children that attended at least one day was 12.6 (SD=10.0) days for the SCV group and 12.3 (SD=10.0) days for the SCV+PI group. In the PI group 10 children attended at least one day of summer programming (i.e., not the intervention summer program) for a mean of 20.3 (SD=3.2) days while 5 children in the control group attended at least one day of summer programming for a mean of 21.6 (SD=4.9) days. For the PI a total of 12 of the 47 (26%) parents completed a goal setting call, 6 in the PI group and 6 in the SCV+PI group. The other 35 parents were unreachable (n=29) or had a phone number that was not in service at the time (n=6). In terms of Fitbit syncing, in the PI group participants synced for a mean of 4.5 (SD=3.5) weeks with 5 participants never syncing, 2 participants syncing 2 weeks, 2 participants syncing 4 weeks, 13 participants syncing 7 weeks, and 1 participant syncing all 8 weeks. For the SCV+PI group participants synced for a mean of 4.8 (SD=3.0) weeks, with 9 participants never syncing, 13 participants syncing for 7 weeks, and 2 participants syncing for 2 weeks.

#### Treatment fidelity.

Treatment fidelity outcomes are also presented in Table 3. The SCV program operated for 6 of the 8 weeks planned. Transportation was never provided for the intervention SCV program. For the PI 44 participants were texted all 7 weeks. Overall, 616 text messages were sent with 518 text messages delivered to the participants. A total of 35 participants received all 14 messages, 1 participant received 13 messages, 1 participant received 10 messages, 1 participant received 5 messages, and 6 participants received 0 messages. Texts were not delivered because 1 participant (who received 5 messages) asked to be removed from the texting list, and 8 participants had a phone number that was not in service.

#### Acceptability.

A total of 14 students completed the satisfaction survey. Results from the survey are presented in Table 4. A total of 89% of children reported that they enjoyed the summer program, with 100% of children reporting that the program was fun.

In the interviews, parents indicated that they were satisfied with the SCV program.

Parent 1: Well, it’s been great. I mean, the teacher’s been good. It’s been, everything’s been on point with the Boys & Girls Club with counselors. They are very supervised, and [child name] seemed to enjoy herself.

Specifically, parents were pleased that the program was free and that it operated at their child’s school.

Parent 2: Also, the school is great, the environment, you know, the location was great.

Parent 3: I didn’t have to pay anything for the program. She got into it for free, and it’s not far from our home.

However, the lack of transportation to the program was a major barrier to participation as indicated by parents and program staff.

Parent 4: Transportation, that was the biggest issue, because their parents don’t drive and it was hard for us to drop him back and forth.

Program leader: And then also, where we were not able to provide that transportation. I know that I talked to a lot of parents, when I called them to see where they were, it was because I didn’t have transportation to get here.

Another issue was the competing programs that the school district was providing due to the CARES act money the district received.

School Principal: …some parents wanted their students to attend here, but because the summer camp and summer school programs were held at other sites, they didn’t want to, like do the transportation back and forth…

B&G Club Area Leader: …the school district did, you know, the four-week summer camp, immediately, and then the four-week summer camp for enrichment. It’s almost like the Golden Corral buffet; do I get the steak or the chicken or do I get the fish. So many options I think some of the parents got confused.

Finally, program staff and the principal indicated that enrollment in all programs was lower than previous years due to COVID-19 which may have led to lower attendance at the SCV program.

B&G Club Area Leader: I think the lack of attendance was an overall [factor], I think the fear of COVID.

Program Leader: One thing that I know that contributed was, of course, the virus COVID-19, that could have potentially impacted student attendance as well.

### Preliminary Efficacy Outcomes

**Figure 2:**
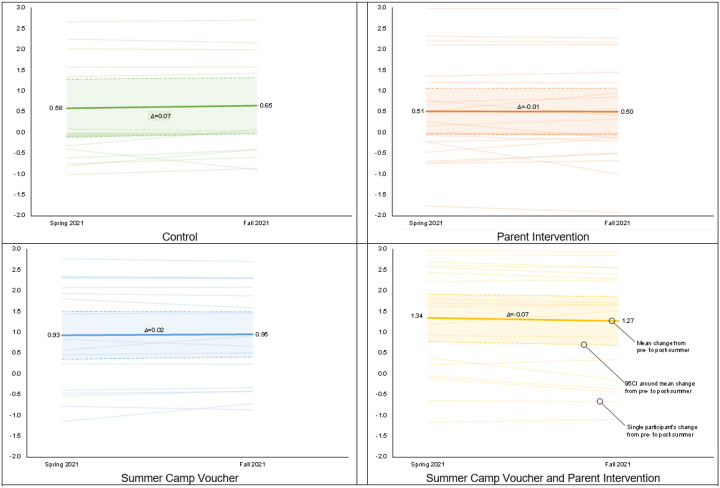
BMI z-score changes by group from pre- to post-summer

Changes in children’s BMI z-score by intervention group are presented in Figure 2a–d. Intent-to-treat analyses showed that no between group BMI z-score changes were statistically significant. The dose response analysis showed that for each day of summer camp programming children attended they gained −0.009 (95CI= −0.018, −0.001) less BMI z-score.

For self-regulation, changes in children’s inhibit, self-monitor, and emotional control t-scores are presented in Table 5. Intent-to-treat analyses showed that no between group changes in inhibit, self-monitor, or emotional control t-scores were statistically significant. Dose response analyses showed that for each day of summer camp programming children attended they experienced a 0.19 (95CI=−0.45, 0.84) and 0.14 (95CI=−0.57, 0.84) greater increase in inhibit and self-monitor scores respectively, but a −0.37 (95CI=−1.19, 0.46) greater decrease in emotional control score. However, none of these differences reached statistical significance.

## Discussion

This study monitored the initial feasibility and preliminary efficacy of SCV and a PI to mitigate accelerated summer BMI gain and improve self-regulation. Feasibility outcomes indicated that operation of the program was impacted by the COVID-19 pandemic and may explain the preliminary efficacy results providing important insight for future interventions. Preliminary efficacy findings indicated that neither intervention nor the combination of the two interventions impacted children’s BMI or self-regulation. However, children that attended for more days experienced less gain in BMI z-score for every day that they attended.

The feasibility metrics for this study were mixed. For instance, the study was able to recruit more participants than originally targeted and parents and children found the program to be acceptable as evidenced by the high enjoyment survey scores and the parents’ satisfaction with the camp from the interviews. Further, retention was high across all intervention groups. However, compliance was low for both the intervention summer camp as evidenced by low attendance and low in the PI as evidenced by the few parents completing a goal setting call, and the low number of weeks syncing their child’s Fitbit. Further, treatment fidelity for the summer camp was poor with the summer program running fewer weeks than planned. However, fidelity was high in the PI with the vast majority of intervention texts delivered.

No statistically significant between-group differences in BMI or self-regulation changes over the summer were observed. This finding is not surprising for two reasons. First, children in the control and PI groups attended summer programming. For instance, 5 children (i.e., 25%) in the control group and 10 children in the PI group (41.7%) attended summer programming. The participating school was initially selected in 2019 because it did not offer any summer programming. However, in the spring of 2020 the school district in which the participating school operated received expanded funding to operate summer programming from the Coronavirus Aid, Relief, and Economic Security (CARES) Act. This funding resulted in the school district operating two additional summer programs that did not exist previously and were free to children in the district. These programs focused on academics and in some instances children who were struggling academically were mandated to attend. Thus, children in the control and PI group had expanded access to summer programming. Second, children in the SCV and SCV + PI did not attend the intervention summer program at the same rate (12.6 & 12.3, days respectively) as children in the control and PI group (20.6 & 20.3, days respectively). This is likely, at least in part, because the school district provided school bus transportation to the district-operated camps while transportation was not provided to the intervention summer camp. Transportation was planned for the intervention camp in this study; however, the district was unable to provide this transportation due to bus driver shortages and the fact that it was providing transportation to the district operated camps. Thus, a lack of differences in between group changes in BMI z-score and self-regulation are likely because of intervention contamination, a large number of children who were not randomized to attend summer camp ended up attending summer camp and many of those randomized to attend summer camp did not. However, it is unlikely that these expanded opportunities to attend summer programming will continue in the future because they were operated with funding from the Coronavirus Aid, Relief, and Economic Security (CARES) Act, a one-time bill that paid for summer programming in the summer of 2020.

While there was no statistically significant between-group difference in change in BMI z-scores the dose response relationship was statistically significant. One previous study that explored the dose response relationship between summer camp attendance and BMI z-score changes found that for each additional day of camp participation children gain − 0.004 (p = 0.06) fewer BMI z-score units over the summer (31, 58). This is similar in magnitude and direction to the current study (i.e., −0.009; 95CI=−0.018, −0.001). However, like the current study, the previous study completed the dose response analysis post-hoc. This finding provides at least partial support for the hypothesis that providing children with access to structured summer programming may mitigate accelerated summer BMI gain. Thus, further studies that test the dose response findings are necessary.

A child’s self-regulatory abilities may also be a key underlying mechanism that is related to a child’s accelerated summer BMI gain (21, 22). This study found no statistically significant differences in changes between groups in children’s ability to self-regulate. However, dose-response analyses showed that children who attended more days of summer programming also trended toward a greater increase in their inhibitory control and ability to self-monitor. While these findings are preliminary, they are suggestive that summer programming may indeed improve a child’s self-regulation which may in-turn reduce accelerated summer BMI gain. This finding is consistent with past research that has shown that the routines embedded within structured days are related to children’s self-regulation (19, 20).

This study has a variety of strengths. First, the study used both quantitative and qualitative measures and methods. This allowed for a comprehensive evaluation of the implementation, feasibility, and preliminary efficacy of the intervention. Second, the study employed a 2×2 factorial design which allowed for the efficient testing of the PI along with the SCV program in a single study. Third, this study was also guided by a theoretical framework the structured days hypothesis. This study also measured self-regulation which may be a key mechanism underlying the structured days hypothesis and accelerated summer BMI gain.

This study must also be interpreted in light of its limitations which include a small sample, operation at a single school, and contamination across groups with children attending summer programming that were not randomized to attend. This study also only collected data on changes in children’s self-regulation and BMI z-score over the summer. Without also collecting further data over the nine-month school year it is impossible to know if these changes represent accelerations in expected changes in these outcomes. Finally, it is critical to understand that this study occurred in the summer of 2021 in the midst of the global COVID-19 pandemic. Thus, the findings need to be carefully interpreted to identify what can be generalized and what is context specific to this unique time period.

Providing children with structured summer programming may be effective for mitigating accelerated summer BMI gain as evidenced by the dose-response findings. However, identifying strategies for increasing attendance at summer programming is critical. One key strategy for doing this may be to provide transportation to and from the summer program. The PI herein was not feasible with low parent engagement. Future interventions should explore strategies targeting increased parent engagement if a parent component is included.

## Supplementary Material

Supplement 1

## Figures and Tables

**Table 1.** Implementation, Feasibility, and Efficacy Evaluation Components and Corresponding Measures

Evaluation Components	Data collection Instruments	Construct	Source	Measurement Frequency
Recruitment Capability	Informed consent forms	The proportion of eligible participants who are enrolled at baseline of the study.	Document Review	Once at baseline
Retention	Summer Day Camp Program Attendance Records	The proportion of enrolled participants who are present throughout the full length of the treatment.	Document Review	Once at follow-up
Compliance	Summer Day Camp Program Attendance Records	Mean number of program days attended.	Document Review	Daily
	Parent Intervention Call and text records	Number of parents completing a goal setting call, and number of weeks syncing their child’s Fitbit	Document Review	Weekly
Efficacy	Height and Weight	BMI z-score change	Children	Pre and post summer
	Teacher Brief	Self-regulation (inhibit, self-monitor, and emotional control subscales)	Children	Pre and post summer
Treatment Fidelity	Summer Day Camp Program Schedule and Attendance Records	Frequency of program delivery (number of program days delivered of the number planned).	Document Review	Weekly unannounced observations
	Program Observation	Duration of program delivery (length of program days delivered out of the length planned)	Research Staff	Four unannounced observations on randomly selected days
	Text records	Frequency of texts delivered (number of texts delivered of the number planned)	Document Review	Weekly
Acceptability	Interviews	Parents satisfaction with Summer Day Camp program and Parent Intervention	Parents	Annual review following HSL program delivery
	Summer Day Camp Enjoyment Survey	Mean rating of program enjoyment	Children	Annual during the last week of the HSL program
	Interviews	Benefits of and barriers to program delivery and attendance	Parents, Administration, Teachers, and Staff	Annual following summer day camp program delivery

**Table 2.** Characteristics of Participants at Baseline

	Control		Parent Only		Summer Camp Only		Parent and Summer Camp Intervention	
Number of Participants	21		24		21		23	
Age (SD)	8.5	(1.7)	8.4	(1.6)	8.0	(1.3)	8.7	(1.4)
Male (%)	45.0		62.5		56.5		50.0	
Race/Ethnicity (%)								
Black	58.3		61.5		52.5		48.2	
Hispanic	29.2		33.9		25.4		28.6	
Caucasian	12.5		4.6		17.0		14.3	
Other	0.0		0.0		5.1		8.9	
Anthropometrics at Baseline (SD)								
Height in ft	4.3	(0.4)	4.4	(0.4)	4.3	(0.4)	4.4	(0.3)
Weight in lbs	75.7	(28.0)	79.9	(33.8)	74.6	(31.7)	91.2	(38.3)
BMI	19.4	(5.5)	19.5	(5.4)	19.3	(5.1)	22.5	(6.9)
BMI z-score	0.61	(1.28)	0.77	(1.26)	0.68	(1.50)	1.29	(1.31)
Self-Regulation at Baseline (SD)								
Inhibit	55.8	(16.0)	56.9	(11.6)	52.7	(15.1)	51.6	(9.0)
Self-Monitor	53.6	(13.8)	55.4	(11.5)	51.3	(12.2)	51.3	(10.2)
Emotional Control	56.8	(17.1)	57.1	(11.8)	56.0	(19.0)	51.8	(11.4)

**Table 3.** Compliance and Fidelity Outcomes by Intervention Group

		Control	Parent Intervention	Summer Camp Voucher	Parent Intervention & Summer Camp Voucher
		21	n=24	n=21	n=23
Compliance	Number of children attending 1 or more days	5	10	7	11
	Mean number of program days attended^3^ (SD)	21.6 (4.9)	20.3 (3.2)	12.6 (10.0)	12.3 (10.0)
	Number of parents completing a goal setting call	-	6	-	6
	Mean number of weeks syncing their child’s Fitbit (SD)	-	4.5 (3.5)	-	4.8 (3.0)
Fidelity	Frequency of program delivery (number of program days delivered of the 40 days planned)			24	24
	Frequency of transportation provided (number of days transportation was provided)			0	0
	Duration of program delivery (hours)	-	-	9.7 (0.24)	9.7 (0.24)
	Number of participants that received all texts	-	19	-	16
	Percent of texts that were delivered	-	85.1%	-	82.4%

a Mean includes children attending at least one day

**Table 4.** Student Report of Summer Camp Voucher Program Acceptability

Q1: When I am in the HSL Summer Program:	Percentage indicating affirmative response
I enjoy it	89%
I feel bored	32%
It’s fun	100%
It gives me energy	83%
It makes me sad	12%
My body feels good	61%
It’s very exciting	95%
It feels good	95%
I want to be doing something else	58%
I enjoy the classroom lessons	79%
I am good at things we do in the classroom	68%
I am good at the games we play in the gym and outside	89%
I am included by others in the classroom	68%
I enjoy playing outside on the playground	100%
I enjoy playing inside in the gym	95%
I enjoy eating breakfast	95%
I enjoy eating lunch	95%
I enjoy eating snack	100%
I enjoy the teachers and staff	83%
I like coming because I made friends	95%
I feel tired	84%
I like the amount of time we spend playing everyday	79%
I like the amount of time spent in the class everyday	83%
I get to decide what I am going to do in the classroom	50%
I get to decide what I am going to do in the gym and outside	58%
I am included by others in the gym and outside	79%

**Table 5.** Changes in self-regulation by group from pre- to post-summer

BREIF^a^ Subscale	Intervention Group	Baseline	SD	Post-intervention	SD	Δ
Inhibit	Control	55.8	(16.0)	62.4	(11.9)	6.6
	Parent Intervention	56.9	(11.6)	60.6	(12.1)	3.8
	Summer Camp Voucher	52.7	(15.1)	51.5	(13.9)	−1.2
	Summer Camp Voucher and Parent Intervention	51.6	(9.0)	56.4	(13.0)	4.8
Self-Monitor	Control	53.6	(13.8)	61.9	(13.4)	8.3
	Parent Intervention	55.4	(11.5)	57.6	(10.0)	2.3
	Summer Camp Voucher	51.3	(12.2)	51.1	(13.7)	−0.2
	Summer Camp Voucher and Parent Intervention	51.3	(10.2)	57.9	(13.2)	6.7
Emotional Control	Control	56.8	(17.1)	55.8	(15.7)	−1.0
	Parent Intervention	57.1	(11.8)	61.1	(16.2)	4.0
	Summer Camp Voucher	56.0	(19.0)	52.1	(14.5)	−3.9
	Summer Camp Voucher and Parent Intervention	51.8	(11.4)	54.9	(15.8)	3.1

a Behavior rating inventory of executive function

## Data Availability

The datasets generated and/or analyzed during the current study are not publicly available due to institutional review board requirements but are available from the corresponding author on reasonable request.

## Key Messages Regarding Feasibility

1. Accelerated summer BMI gain may be mitigated by structured summer programming. However, little is known about the feasibility of providing children with access to vouchers to attend structured summer programming. Further, self-regulation is a key construct for maintaining a healthy weight during the summer, but little is known about the feasibility of targeting children’s self-regulation through a combination of structured summer programming and a parenting intervention to mitigate accelerated summer BMI gain.
2. This pilot demonstrated that providing children with structured summer programming may be effective for mitigating accelerated summer BMI gain as evidenced by the dose-response findings. However, identifying strategies for increasing attendance at summer programming is critical. One key strategy for doing this may be to provide transportation to and from the summer program. The parent intervention herein was not feasible with low parent engagement. Future interventions should explore strategies targeting increased parent engagement if a parent component is included.
3. Findings from this study will be used to improve the summer voucher program (e.g., provide transportation to programming) and parent intervention (i.e., increase parent engagement).

## Abbreviations

SCV: summer day camp voucher
PI: parent intervention
SCV+PI: summer day camp voucher and parent intervention
MVPA: moderate-to-vigorous physical activity
BMI: body mass index
